# A Narrative Review of Loneliness and Brain Health in Older Adults: Implications of COVID-19

**DOI:** 10.1007/s40473-021-00237-6

**Published:** 2022-06-11

**Authors:** Janelle N. Beadle, Angela Gifford, Abi Heller

**Affiliations:** 1 Department of Gerontology, University of Nebraska at Omaha, 6001 Dodge Street, CPACS Room 211, Omaha, NE USA; 2 Department of Psychology, Neuroscience and Behavior Graduate Program, University of Nebraska at Omaha, Omaha, NE USA; 3 Department of Neurological Sciences, University of Nebraska Medical Center, Omaha, NE USA

**Keywords:** Loneliness, Social isolation, Older adulthood, Aging, Neuroimaging, COVID-19

## Abstract

**Purpose of Review:**

This narrative review highlights important factors contributing to loneliness in older adults prior to and during the COVID-19 pandemic and effects on brain health.

**Recent Findings:**

We characterize risk factors for loneliness in older adulthood and the impact of COVID-19. Furthermore, we discuss the implications of loneliness for older adults’ brain health.

**Summary:**

Understanding the multifactorial causes of loneliness in different subpopulations of older adults both before and during the COVID-19 pandemic will provide insights for the development of interventions targeted to reduce loneliness in older adults based on their specific risk factors.

## Introduction

Before the coronavirus 19 (COVID-19) pandemic, social isolation and loneliness in older adult populations were already a significant public health concern, with up to 39% of community-dwelling older adults experiencing loneliness [1]. In fact, loneliness and social isolation are serious public health issues because of the increased risk for numerous physical and mental health issues, such as depression, cardiovascular disease, cognitive decline, and Alzheimer’s disease, in addition to being associated with more physician visits, and greater mortality risk [2–12]. Due to COVID-19, concerns escalated for the older adult population, as many found themselves becoming increasingly isolated as they attempted to reduce COVID-19-related health risks. The primary purpose of this narrative review is to discuss our current understanding of the psychological and neural bases of loneliness in older adulthood, and the degree to which specific demographic, personality, and environmental factors increase older adults’ risk for loneliness. Furthermore, we investigate the impacts of the COVID-19 pandemic on older adults’ psychological health and risk of mental health issues and discuss implications for brain health. In the first section, we will define loneliness and describe key differences between loneliness and the related concept of social isolation.

### Factors Associated with Loneliness Across the Adult Lifespan

Although the terms social isolation and loneliness are often used interchangeably in the popular press, in a scientific context there are critical differences between these two concepts [13]. Specifically, social isolation is defined as having few social contacts and reduced social connections [14]. It is typically measured objectively by direct, numerical assessment of the total number of social connections within one’s network, in addition to the frequency with which an individual interacts with others and participates in community activities [14]. On the other hand, loneliness is defined as the individual’s perception of frequently being alone and a dissatisfaction with one’s social network size and/or quality, regardless of the specific size of the network [13]. Therefore, loneliness is different from social isolation because it is a perception about one’s current situation rather than an objective assessment of one’s frequency of social interaction. Certain demographic factors may increase one’s risk for loneliness, including relationships of poor quality, not being married, and/or lower socioeconomic status [15].

An important factor when considering age group differences in loneliness is that there are key individual differences in personality and emotional reactivity between individuals that can contribute significantly to loneliness, regardless of age. Individuals with higher loneliness tend to also have higher levels of a personality factor called neuroticism which is a general tendency to experience negative emotion, anxiety, or distress in daily life [16, 17]. This may influence how individuals view their social interactions with others.

To better understand the experience of loneliness in older adulthood, it is critical to comprehend how loneliness levels can change across the adult lifespan. There is growing evidence that late adolescence/early adulthood is a time when loneliness levels peak [18, 19]. Although much of adulthood is characterized by lower levels of loneliness, rates tend to increase in the oldest-old, typically individuals approximately 75–80 years of age and older [1, 18]. The estimated prevalence of loneliness in older adults ranges between 20–34% percent in the community, with even higher levels in long term care settings [1, 20–23].

### Loneliness in Older Adulthood

#### Age-related Demographic and Relational Shifts That Impact Loneliness

Importantly, as people age, there is a normal winnowing of social network size, often to include only a handful of very close others, such as friends and family [24, 25]. Yet, despite the decreased size of their social networks, there is evidence that older adults report feeling greater emotional well-being than younger adults with much larger networks [24, 25]. The observation that older adults have smaller social networks than younger adults is consistent with the socioemotional selectivity theory which purports that older adults prioritize activities and people that bring them greater emotional meaning [24]. Although older adults may be content with their shrinking social networks, this may put them at greater risk for experiencing social isolation and loneliness over time (see Table 1).

**Table 1:** Loneliness in older adults, review of key findings

Authors	Participants	Measurements	Analyses	Findings	Disease/Risk Factor
Neurotypical Older Adults					
Cacioppo et al., (2002) [3]	Study 1: 89 young adults, *M*_age_ = 19.26 yr, age range 18–24 yr Study 2: 25 older adults, *M*_age_ = 65 yr, age range 53–78 yr	R-UCLA Loneliness Scale, depression (BDI-S), sleep quality (PSQI), salivary cortisol assays, cardiovascular activity, exercise measure, health behavior survey, verbal mental arithmetic, speech tasks, orthostasis stressor	Correlation, ANOVA	Lonely older adults have poorer sleep quality and greater age-related blood pressure increases than non-lonely older adults	
Düzel et al., (2019) [26]	319 older adults, ages 61–81 yr, *M*_age_ = 70.0 yr, *SD*_age_ = 3.7	sMRI—VBM; UCLA Loneliness Scale	structural equation modeling, latent hierarchical regression analyses	Higher loneliness associated with smaller gray matter volume in left amygdala/anterior hippocampus, left posterior parahippocampus and left cerebellum	
Gerst-Emerson & Jayawardhana, (2015) [5]	3,530 older adults, age range 60–100 yr; Data from: 2008 and 2012 Health and Retirement Study	Three-Item Loneliness Scale, depression measure (CES-D), health care utilization, objective and subjective health, Activities of Daily Living Scale, financial satisfaction	Panel negative binomial regression method, sensitivity analysis	Chronic loneliness was significantly, positively associated with the number of physician visits	
Lan et al., (2016) [27]	85 male participants, 65 years and older (M_age_ = 80.3 yr)	depression measure (GDS), UCLA Loneliness Scale Version 3, functional connectivity density mapping	Regression	Positive correlation between short-range functional connectivity density and loneliness scores in the bilateral lingual gyri of the brain	
Luo et al., (2012) [7]	2,101 adults age 50 yr and older from the Health and Retirement Study 2002 and 2008 waves	Revised UCLA Loneliness Scale, mortality (National Death Index), depressive symptoms (CES-D), self-rated health, functional limitations	Longitudinal study design, correlations, survival analysis, hazard models, cox models, cross-lagged path analysis	Loneliness was associated with greater mortality risk over 6 years; loneliness affected functional limitations and depressive symptoms	
Yang et al., (2018) [23]	2,770 older adults 60 yr and older, M_age_ = 69.72 yr, SD_age_ = 8.07	De Jong Gierveld six-item scale for loneliness, self-rated health, IADL, emotional health of family (General Functioning Scale of the McMaster Family Assessment Device), frequency of socializing with neighbors, new media use	Correlation, regression of whole sample, regression split by age groups of young old (60–79) and oldest old (80+)	Overall, older adults with higher loneliness were single and divorced, education less than high school, living alone, poorer health and family functioning, less frequent socializing, less volunteerism, less frequent new media use; In oldest-old group, living arrangement, self-reported health and family functioning remained significant among these other factors	
Associations with Disease States					
Christiansen et al., (2016) [2]	8,593 older adults, age range: 65-103 yr	2013 Danish Public Health Survey “How are you?”, Danish version of the Three-Item Loneliness Scale, measure of chronic diseases (e.g., cardiovascular disease, diabetes, and migraine), Perceived Stress Scale, measure of health behaviors (daily smoking, alcohol problems, weekly physical activity, and dietary habits), measure of sleep quality	Mediation analyses	Significant association between loneliness and cardiovascular disease, diabetes, and migraine; Factors including high perceived stress, physical inactivity, daily smoking, and poor sleep, mediated association between loneliness and health conditions	Cardiovascular disease, migraines, and diabetes
Hawkley et al., (2010) [6]	229 older adults, 50-68 yr at study onset, M_age_ = 57.4 yr, SD_age_ = 4.5, from Chicago Health, Aging, and Social Relations Study, tested annually for 5 years	Systolic blood pressure, UCLA Loneliness Scale–Revised, demographic covariates, smoking status, alcohol consumption, BMI, physical activity, cardiovascular medications, chronic health conditions, depressive symptoms (CES-D), Perceived Stress Scale, Cook-Medley Hostility Scale, Interpersonal Support Evaluation List	Correlation, cross-lagged panel analyses	Loneliness at baseline predicted increased systolic blood pressure at Year 2, 3, and 4; effect was independent of age, gender, race or ethnicity, cardiovascular risk factors, medications, health conditions, depressive symptoms, social support, perceived stress, and hostility	Cardiovascular risk factors
Rafnsson et al., (2020) [11]	6,677 older adults, at baseline age range 52–90 yr, *M*_age_ = 66 yr, *SD* = 9.4	Three-item short form of Revised UCLA Loneliness Scale, dementia assessment, memory (immediate and delayed recall), time orientation, index measuring extent of social contact	Longitudinal, Cox proportional hazards regression models	Dementia risk positively related to higher loneliness and negatively related with number of close relationships and being married	Dementia
Sundström et al., (2020) [9]	1,905 older adults 60+ years of age at baseline, follow-up time of up to 20 years	Single item measure of loneliness, “Do you often feel lonely?”, dementia diagnosis assessment, MMSE, depressive symptoms (CES-D)	Longitudinal, multivariate-adjusted Cox proportional hazard models	Participants who reported higher levels of loneliness at baseline had increased risk of developing dementia over the course of the study	Dementia, Alzheimer’s Disease
Theeke & Mallow, (2013) [8]	60 chronically ill older adults	UCLA Loneliness Scale Version 3, quality of life measure (CASP-12 Scale), assessment of chronic illness	Descriptive and Correlational	Individuals with a mood disorder had highest average loneliness scores; loneliness correlated with number of chronic illnesses and benzodiazepine use	Hypertension, hyper-lipidemia, heart disease, arthritis, diabetes, lung disease, mood disorders, stroke, cancer, and obesity
Wong et al., (2016) [28]	31 older adults diagnosed with late life depression, Mage = 67.45 yr, SDage = 5.43, 23 healthy comparison participants, Mage = 67.13 yr, SDage = 4.79	neuroimaging (DTI, task-based fMRI affective processing task), UCLA Loneliness Scale, depressive symptoms (HAMD)	t-tests, ANOVA, regression	Positive association between functional connectivity in the default mode network and corticostriatal network in individuals with late life depression when viewing negative stimuli	Late life depression
Zhong et al., (2017) [12]	14,199 older adults in China, age range 65–105 yr at baseline, *M*_age_ = 85 yr, *SD* = 11.32	Single loneliness question, Chinese version MMSE, chronic condition checklist	Longitudinal, latent variable cross-lagged panel analysis	Severe loneliness predicted poorer cognitive function at later time-points and vice versa; Partial mediation by change in number of chronic conditions	Hypertension, diabetes, heart disease, stroke and other cerebrovascular diseases, cancer, arthritis, dementia

*BDI-S*, Beck Depression Inventory-Short Form; *CASP-12*, Quality of Life Index in Older Adults; *CES-D*, Center for Epidemiologic Studies Depression Scale; *DTI*, Diffusion Tensor Imaging; *fMRI*, functional magnetic resonance imaging; *GDS*, Geriatric Depression Scale- short form; *HAM-D*, Hamilton Rating Scale for Depression; *HCP*, Health Care Practitioner; *IADL*, Instrumental Activity of Daily Living; *MMSE*, Mini-Mental State Exam; *PSQI*, Pittsburgh Sleep Quality Index; *sMRI*, structural magnetic resonance imaging; *VBM*, Voxel-Based Morphometry; *yr*, years of age

Important risk factors for loneliness include having a small social network size, or few high-quality social contacts [15, 18]. Older adults, on average, have smaller social networks than younger adults [25, 29]. Furthermore, the composition of older adults’ social networks also differs from younger adults, as it is typically made up of close friends and family members, rather than the broader social networks of acquaintances found in younger adulthood [25, 29]. There are thought to be multifactorial causes for the changes in social networks in older adulthood which can include demographic shifts, health changes that result in functional limitations, role changes, and motivational changes, among other factors [25, 29].

Demographic factors that impact older adult networks can include the death of close friends or family members due to advanced age, or declining health that results in functional limitations, and may reduce likelihood of social interactions [7]. Older adults may also develop health issues that lead to functional changes, limiting their ability to participate in community events [7]. There are also role changes in aging. Many older adults are retired which means they miss out on the workplace social contacts that are typical of younger and middle adulthood. Furthermore, older adults typically no longer have children in their home, and thus do not have as many opportunities for social engagement with the parents of their children’s friends that they might have had in younger and middle adulthood. For these reasons and other relevant factors, many older adults experience lower levels of social integration (i.e., a measure of one’s social network size, social support, and engagement) [30]. Lower levels of social integration in older adults are also associated with greater loneliness [30].

#### Changes in Relational Motivation

Older adults’ declining social network sizes may be motivated by specific factors, in particular a desire to have emotionally meaningful and positive relationships with close others. This concept is described in the socioemotional selectivity theory which purports that because older adults realize their time is limited, they tend to focus on experiences that bring them greater emotional meaning [24]. This may help to explain why older adults winnow their social networks to the few people who bring them joy and with whom they experience the most emotional meaning.

This motivational shift may also help to explain why, on average, older adults living independently typically do not report higher levels of loneliness than middle-aged adults. However, during the COVID-19 pandemic, this dynamic shifted because community-dwelling older adults experienced restrictions in their ability to spend time with their close others in person due to health-related pandemic concerns. Due to older adults’ motivation to foster experiences with high levels of emotional meaning, providing access to close others may reduce loneliness to a greater degree than time spent with new acquaintances.

#### Older Populations in Long-Term Care Facilities

There have been growing concerns of increased loneliness in older adult populations who reside in nursing homes and long-term care facilities [21, 31]. One study examined the prevalence of loneliness in long-term care facilities in Finland from 2011 to 2017 and found that approximately 36% of the sample was lonely, with stable prevalence rates over time [21]. A study based in Norway reported even higher rates of loneliness (56%) in nursing home residents 65 years and older who did not have a cognitive impairment [20]. For individuals living in long-term care facilities, the rates of high loneliness (22–42%) were significantly greater than for individuals living independently (10%) [31, 32]. Furthermore, older individuals with the highest levels of loneliness were more likely to be admitted to a nursing home within 4 years than those who were less lonely [33]. Loneliness was also associated with admission into a care home, even after accounting for other typical predictors of loneliness (e.g., age, sex, social isolation, and depression) [34].

Researchers have begun to examine the mechanisms for greater loneliness levels of older adults in nursing homes in comparison to community-dwelling older adults [35]. Relevant factors associated with greater loneliness that may contribute to placement in a nursing home include poor health and more functional and cognitive issues that may reduce their ability to interact socially with others [36]. For many living in these communities, long-term care facilities attempt to reduce loneliness by organizing community events, social engagements, and communal dinners that encourage interaction between residents, but many of these social activities were canceled due to the COVID-19 pandemic.

## Implications of Older Adult Loneliness for Brain Health

Loneliness is a risk factor for multiple physical and mental health conditions in older adulthood [2, 37–39]. In particular, loneliness can have significant effects on brain health, affecting mental health, cognition, and risk of dementia [8–12, 37, 39]. Older adults with higher levels of loneliness are more at risk for developing depression and have poorer long-term depression outcomes [39]. Furthermore, the combination of depression and loneliness predicts more metabolic risk than depression alone, especially in women [38]. Loneliness can also increase older adults’ risk for cognitive issues [37]. For instance, in a sample of 8,030 older adults 50 years and older from the U.S. Health and Retirement Study, the authors found that baseline loneliness was associated with higher levels of cognitive decline over a 12-year period, even after accounting for depression, health, social network size, and multiple demographic factors (e.g., age, sex, education, socioeconomic status) [37]. Loneliness is a significant risk factor for Alzheimer’s disease and related dementias [9, 10]. To better understand potential psychological and brain mechanisms by which loneliness is associated with poorer brain health outcomes in older adulthood, we will describe our current understanding of the brain correlates of loneliness in adulthood.

### The Neural Correlates of Loneliness and Implications for Brain Health

Researchers have primarily examined the brain correlates of loneliness through structural and functional magnetic resonance imaging methods (sMRI, fMRI) [4, 26, 28, 40–42]. A study of loneliness in young adults examined associations between white matter and loneliness scores on the UCLA loneliness scale [41]. The researchers found a negative correlation between loneliness scores and regional white matter density in regions typically associated with social cognition, such as the left posterior superior temporal sulcus, right anterior insula, posterior temporoparietal junction, and dorsomedial prefrontal cortex, among other regions [41]. Another research group found that lonelier individuals had less gray matter in the left posterior superior temporal sulcus [40].

Early studies of younger adults used fMRI tasks focused on a construct related to loneliness, social exclusion, rather than measuring loneliness directly [43, 44]. Social exclusion is the perception of being avoided or ignored in a social context which is relevant to the understanding of loneliness because lonely individuals tend to have an increased sensitivity to social threats in the environment and may interpret social exclusion as a consequence of their own stable, negative personal characteristics, rather than temporary environmental situations [16, 43, 45]. Social exclusion is typically measured through a task called, “Cyberball,” in which participants engage in a virtual ball tossing game with two other virtual players and experience social exclusion when the other players no longer include them in the game [43]. In task-based fMRI studies, when participants are excluded in the game, they generally recruit key regions involved in processing social information about the self and others, such as the medial and ventrolateral prefrontal cortex and the anterior cingulate cortex (ACC) [43, 46].

A small number of studies have specifically examined the neural bases of loneliness using task-based fMRI studies. These studies have assessed lonely individuals’ reactions to social versus non-social images, often from the International Affective Picture System (e.g., an example of a social, pleasant image is a photo of man and dog running together) [47], or by showing images of a close other [48]. In response to unpleasant images, lonely younger adults showed greater activation in the visual cortex to images of people than objects [47]. There have been mixed findings about the role of the ventral striatum for loneliness, with one study finding less activation in lonely than non-lonely individuals [47], and another study finding that loneliness was associated with more activation [48]. These mixed results may be due to differences in the type of stimuli used, such as the images of strangers [47], versus images of close others [48].

Neuroimaging studies examining loneliness have also investigated intrinsic brain networks associated with loneliness using resting-state functional connectivity analyses (rs-FC). Studies using this fMRI analysis technique have uncovered altered connectivity patterns in cingulo-opercular, limbic, default, and dorsal and ventral attention networks in lonely and depressed adolescents and adults [42, 49,50,51,52•,^53^]. Furthermore, in a large sample of 942 adults researchers found that individuals with higher levels of loneliness have less modular connections between specific brain networks, including the default, frontoparietal, attention, and perceptual networks [52•]. In younger adults, loneliness was associated with greater functional connectivity between key node areas within the cingulo-opercular network, such as the insula and anterior cingulate cortex [51]. These studies suggest the possibility that brain regions important for processing emotion and reward and thinking about the self versus others may be impacted in younger adults who are lonely.

### Neural Correlates of Loneliness and Social Isolation in Older Adulthood

A small number of studies have investigated structural brain differences related to loneliness in older adulthood [26, 28, 54]. There is evidence that lonelier older adults have smaller white matter volumes than less lonely older adults [54]. Another structural MRI study showed that older individuals with higher levels of loneliness have smaller gray matter brain volumes than less lonely individuals in key regions of the medial temporal lobe important for emotional experience and memory (e.g., the left amygdala, hippocampus, and posterior parahippocampus) [26]. Another study investigated structural and functional brain networks associated with loneliness in older adults with late life depression and found that loneliness was linked to connectivity between the superior frontal gyrus and amygdala region [28].

Researchers have also examined differences in functional brain activity through task-related fMRI studies in response to social isolation and loneliness in younger and older adults [55,56•]. One study compared younger versus older adults on a fMRI task examining social perception in response to scenes of individuals engaged in affiliative behaviors (e.g., groups of 2 or more people interacting), depicted in an isolated context (e.g., lone individuals), and a non-social context (e.g., objects or natural scenes) [55]. This study found that both age groups showed greater brain activity to social scenes than non-social scenes in the temporal pole, mPFC, and amygdala, similar to the other studies we have described in younger adults. One of the key age-related findings was that younger adults showed greater activity in the hippocampus than older adults when perceiving social versus non-social scenes.

In this study, there were differential age-related responses to affiliative versus isolation scenes in two regions: the precuneus and the temporal pole. Specifically, in the precuneus, older adults showed a greater response to affiliative scenes than scenes of isolation, with the reverse finding in younger adults. In the temporal pole, younger adults showed greater activity to affiliative then isolation scenes, with older adults responding more to scenes of isolation. In summary, this study found that there were age-related differences related to processing social isolation in the precuneus and temporal pole, and that the hippocampus was recruited in general for the processing of both affiliation and isolation contexts.

A recent study with a large sample of younger and older adults examined the relationship between participants’ responses to social versus non-social scenes, but did not find any significant relationships between brain activity to social scenes as a function of loneliness in two a priori regions of interest, the amygdala and ventral striatum [56•]. Because the study did not examine the whole brain, it is not known whether there were differences in other brain areas. Other studies have investigated intrinsic functional brain connectivity patterns associated with loneliness in middle-aged and older adults [27, 57]. One study using the UK Biobank population investigated adults who were 40-69 years of age at baseline and found that lonelier individuals had a higher level of functional connectivity within the default network [57], a network often implicated in self-referential thinking, as well as social processing.

In summary, there is growing evidence that loneliness is associated with both structural and functional brain differences in younger and older adulthood. Structural neuroimaging studies point to decreased white and gray matter volume in structures that play critical roles in processing social information about the self and others, as well as memory for socioemotional information. Similarly, task-based and resting-state fMRI studies point to reduced brain activity in and between networks that support social cognition and understanding others’ mental states and emotions. Taken together, there is preliminary evidence from studies of younger and older adults demonstrating that loneliness is associated with individual differences in structure and function in key brain regions linked to social processing.

Despite loneliness already being a significant concern prior to the COVID-19 pandemic, it may be that during the pandemic certain subgroups of older adults may have experienced even higher loneliness which in turn may have negatively affected their brain health. In particular, the brain-related differences typically seen in lonely individuals may have been exacerbated by the high levels of loneliness and social isolation experienced during the pandemic. Thus, it is possible that both the structural and functional brain differences associated with loneliness in older adulthood may have been even more pronounced during the peak of the COVID-19 pandemic. Future studies should examine this question directly, by conducting longitudinal brain imaging studies that investigate brain changes prior to, during, and post-pandemic in older adulthood.

### Loneliness During the COVID-19 Pandemic

Older adults are at greater risk of experiencing collective isolation in which an individual develops loneliness from a perceived lack of connection with the greater community. During the COVID-19 pandemic, many older adults experienced an exacerbated form of collective isolation not only from COVID-19 but also from the media, as some older adults perceived messages from the media of “not mattering” that further increased the psychological divide between older adults and other age groups [58, 59]. Typically, perceptions of collective isolation may be reduced by involvement with community groups and volunteer positions, but COVID-19 limited access to these programs, as many were closed temporarily for safety concerns [58, 60]. However, technological interventions such as virtual reality and video calls are being developed to combat this issue and have shown initially promising results [61].

Researchers found loneliness to be one of the main risk factors for depression and anxiety in older adults during the pandemic [62, 63]. Interestingly, a study by Bu and colleagues [64•] found that risk factors linked to loneliness were similar both before and during the pandemic [64•]. A few factors that were already associated with loneliness risk prior to the pandemic were linked to even greater risk during the pandemic. In particular, younger adults between the ages of 18 to 30 had greater loneliness than older adults (60 years and older) living independently both before and during the pandemic. Other risk factors that predicted even greater loneliness during the pandemic were living alone and having a low household income. One factor that showed an elevated risk for loneliness during the COVID-19 pandemic was being a current student, perhaps because during the pandemic, many students had to acclimate to remote classes and fewer in-person interactions. In summary, these study results suggest that both before and during the COVID-19 pandemic, younger adults were more at risk for loneliness than older adults living independently. However, it is important to note that these results may not generalize to all older adults, as this sample consisted of predominantly community-dwelling, independently living older adults, rather than older adults in assisted living or nursing home settings.

Thus far, results are inconclusive about whether loneliness increased in community-dwelling older adults living independently during COVID-19, and levels may have fluctuated throughout the pandemic [64•,^65^,^66^]. A key consideration in understanding loneliness levels in older adults is characterizing the degree to which they live independently or in an assisted living context with or without close others (e.g., a spouse), as this provides additional information about their social supports, social access, and daily activities [23]. In this context, it is important to consider that older adults of different ages or living arrangements may experience varying levels of loneliness, with those at the highest risk for loneliness including adults 80 years of age and older, older adults living alone, or living in a long-term care facility [23, 64•, 67]. During the COVID-19 pandemic, researchers found that older adults that perceive themselves to be older, irrespective of their actual age, may be at risk for greater psychiatric symptoms associated with loneliness than those who perceive themselves to be younger [63]. For the experience of late-life depression, or depression onset in older adulthood, loneliness may be a key contributing factor [39].

Due to COVID-19, in most locations, social events in long term care settings were suspended due to health concerns for the residents [59]. Research on the direct impact of loneliness in assisted living and nursing homes was limited during COVID-19. This is due in part to the preferred methodology for collecting data with high-risk populations being online surveys, a format that may be less accessible for older adults in assisted living and nursing homes [68].

#### Implications and Future Directions

Although already a serious public health concern, loneliness became an even greater issue during the COVID-19 pandemic, as individuals at risk prior to COVID-19 experienced even higher levels of loneliness. Furthermore, older individuals in long-term care may have been especially at risk for elevated loneliness due to the COVID-19-related restrictions on social interaction which limited interactions within the facility among residents, and outside visits from family members. To help combat loneliness in older adults, it is relevant to consider the degree to which the causes are situational (e.g., isolation induced by the pandemic), demographic (e.g., being single), or ongoing risk factors, such as a personality tendency towards neuroticism. By identifying the cause of loneliness in the individual, tailored interventions can be developed to specifically address individual risk factors. The choice of intervention may also vary based on resources available in a specific setting, such as access to technology. Therefore, different modes of intervention delivery are needed in order to fit the needs of community members living independently versus individuals who receive assistance or live in long-term care facilities. Although a variety of interventions for loneliness in long-term care settings have already been examined, such as laughter therapy, horticultural therapy, and reminiscence therapy [69], more research is needed to identify robust, effective, and generalizable interventions. In particular, carefully controlled clinical trials are needed to determine which loneliness interventions are most effective depending on the specific type of loneliness-related risk factor.

## Conclusions

This narrative review highlighted key factors linked to loneliness in older adults prior to and during the COVID-19 pandemic. Furthermore, this review highlighted subgroups of older adults who may be at the most risk for experiencing loneliness, such as older adults living in long-term care facilities and individuals older than 80 years of age. Finally, we discussed relationships between loneliness and brain health, and the implications of COVID-19. Taken together, because the mechanisms of loneliness are multifactorial and may vary by situational, demographic, and individual difference factors, it may be useful if interventions designed to remediate loneliness account for the specific combination of risk factors affecting the individual.

## Declarations

### Open Practices Statement

This paper is a narrative review. None of the data or materials for the experiments reported here is available, and none of the experiments was preregistered.
